# Head and neck cancer burden in India: an analysis from published data of 37 population-based cancer registries

**DOI:** 10.3332/ecancer.2023.1603

**Published:** 2023-09-21

**Authors:** Sonali Bagal, Atul Budukh, Jarnail Singh Thakur, Tapas Dora, Burhanuddin Qayyumi, Divya Khanna, Dolorosa Fernandes, Priyal Chakravarti, Ravikant Singh, Suvarna Patil, Rajesh Dikshit, Pankaj Chaturvedi

**Affiliations:** 1 Centre for Cancer Epidemiology (CCE), Advance Centre for Treatment, Research and Education in Cancer (ACTREC), Tata Memorial Centre (TMC), Navi Mumbai 410210, India; 2 Homi Bhabha National Institute (HBNI), Training School Complex, Anushakti Nagar, Mumbai 400094, India; 3 Post Graduate Institute of Medical Education and Research (PGIMER), Chandigarh 160012, India; 4 Homi Bhabha Cancer Hospital (HBCH), Tata Memorial Centre (TMC), Sangrur 148001, India; 5 Homi Bhabha Cancer Hospital and Research Centre (HBCH&RC), Tata Memorial Centre (TMC), Muzaffarpur 842004, India; 6 Mahamana Pandit Madan Mohan Malaviya Cancer Centre (MPMMCC), Tata Memorial Centre (TMC), Varanasi 221005, India; 7 Homi Bhabha Cancer Hospital and Research Centre (HBCH&RC), Tata Memorial Centre (TMC), Visakhapatnam 530053, India; 8 Bhaktshreshtha Kamalakarpant Laxman Walawalkar (BKLW) Hospital, Diagnostic and Research Centre, Ratnagiri 415606, India; a https://orcid.org/0000-0002-2510-1751; b https://orcid.org/0000-0001-6723-802X; c https://orcid.org/0000-0002-8270-4268; d https://orcid.org/0000-0001-7856-8059; e https://orcid.org/0000-0003-2163-796X; f https://orcid.org/0000-0003-4830-0486; g https://orcid.org/0000-0002-3520-1342

**Keywords:** head and neck cancer, mouth neoplasm, oropharyngeal neoplasms, hypopharyngeal neoplasm, laryngeal neoplasms, incidence, registry, India

## Abstract

Head and neck cancer (HNC) is a major public health problem in India. This article presents the HNC burden in different regions of India. The published population-based cancer registries (PBCRs) data from the National Cancer Registry Programme, Bengaluru, and the Tata Memorial Centre, Mumbai, India, were utilised. The 37 PBCRs were divided into six regions including central, east, north, northeast, west and south. The age-standardised incidence rate of HNC was 25.9 (95% CI 25.7–26.1) and 8.0 (95% CI 7.9–8.1) per 100,000 population, respectively, in males and females. HNC accounted for about 26% of all cancer cases in males and 8% in females. The risk of developing HNC was 1 in 33 for males and 1 in 107 for females. The northeastern registries reported the highest incidence rate 31.7 per 100,000 population in males followed by northern (28.5), central (28.3), western (24.4), southern (23.9) and eastern (18.3). In females, the incidence was in the range of 6.2–10.1 per 100,000 population. For all PBCRs together, the HNC burden was two to three times higher in the age group 60+ as compared to 20–39 years. The HNC burden in India is higher than in the USA, UK, Australia, Africa and Brazil. The PBCRs from the south-east Asia region such as the Colombo district, Sri Lanka, as well as Siraha, Saptari, Dhanusha and Mohattari – Nepal have also reported a high burden of HNC. All regions reported mouth as a leading cancer site followed by tongue, larynx, hypopharynx and tonsil except the northeastern region registries where hypopharynx was the top leading cancer. The burden of other sites of HNC is low. Raising awareness of the disease and associated risk factors, providing early detection services, as well as easy access to diagnosis and treatment are required. The government should focus on building the infrastructure and capacity building to control this disease.

## Introduction

In India, the number of cancer cases is rising. According to GLOBOCAN 2020, there will be 2.1 million new cancer cases in India by 2040, an increase of 57.5% from the year 2020 [1–3]. Moreover, one in nine Indians has a lifetime risk of developing cancer [3]. Head and neck cancer (HNC), in particular, accounts for 30% of the all-cancer cases [4]. Furthermore, a significant rise in the incidence of HNC was noted in the Indian population-based cancer registries (PBCRs) of Aurangabad, Delhi, Chennai, and Bhopal for males, and Nagpur for females [5]. HNC includes the cancers of the oral cavity, pharynx, and larynx – primarily originating from the mucosal epithelium, but also from less common sites like salivary glands, and nose, sinuses, muscles and nerves [6]. Squamous cell carcinoma makes up most cases of HNC [7].

In India, more than 65% of patients with HNC attend the hospital with locally advanced disease [8–10]. Late-stage presentation, lack of access to cancer care and failure to complete treatment lead to poor survival in HNC patients [11, 12]. High-quality data from PBCRs are required to address this public health issue as well as to make better infrastructure and policy-related decisions about disease prevention. However, in India, PBCRs cover less than 15% of the population [3, 13, 14]. Several challenges have been reported in establishing PBCRs in resource-constrained countries like India [15, 16].

This research paper aims to summarise the region-wise incidence of HNC based on the published data of the registries of that region. The study utilised the published and publicly available Indian PBCRs data. Data from 28 PBCRs available in public domain of the National Cancer Registry Programme (NCRP), Bengaluru [5] and 9 PBCRs established by the Tata Memorial Centre (TMC), Mumbai (for the states of Maharashtra, Punjab, Uttar Pradesh, Bihar, and Andhra Pradesh as well as the Chandigarh-Union Territory) were included [17–25]. The Barshi registry was the country’s first rural cancer registry, established by TMC, Mumbai in 1987 and regularly submitted data to NCRP.

There is a variation in the definition of HNC by the National Cancer Institute and the International Agency for Research on Cancer (IARC) [26]. Therefore, there is a need to specify the anatomical subsites covered in this study. Here, we have defined HNC based on the International Classification of Diseases (ICD-10) topography codes which include lip (C00), tongue (C01-02), mouth (C03-06), tonsil (C09), other oropharynx (C10), nasopharynx (C11), hypopharynx (C12-13), pharynx unspecified (C14) and larynx (C32). The cancers of salivary gland, maxillary sinus and nasal cavity are not included in the present data due to the different etiology of the disease.

## Method

As per the geographical area, the 37 PBCRs were categorised into six regions including central, eastern, northern, northeastern, southern and western India. The central region included Bhopal, Nagpur and Wardha PBCRs (year 2012–2016). The eastern region included Kolkata PBCR (year 2012–2015). The northern region included Chandigarh, Delhi, Mansa, Patiala, Sangrur, SAS Nagar, Varanasi and Muzaffarpur PBCRs (year 2012–2019). Cachar, Dibrugarh, Kamrup urban, Manipur state, Meghalaya, Mizoram state, Nagaland, Pasighat and Sikkim state, Tripura state and West Arunachal PBCRs are included under the northeast region (year 2012–2016). The southern region included Bengaluru, Chennai, Hyderabad, Kollam, Thiruvananthapuram and Visakhapatnam PBCRs (year 2012–2018). The western region included Ahmedabad urban, Aurangabad, Barshi rural, Mumbai, Osmanabad-Beed, Pune, Ratnagiri and Sindhudurg (year 2012–2018). The registry data available in the public domain of NCRP are for the year 2012–2016 except for Bhopal, Kolkata, Mumbai, Osmanabad-Beed (2012–2015), Delhi, Bangalore (2012–2014) and Hyderabad registries (2014–2016). The TMC PBCRs included data for the period 2017–2018 of Chandigarh, Sangrur, SAS Nagar, Mansa, Visakhapatnam, Ratnagiri and Sindhudurg cancer registries. Data for Varanasi and Muzaffarpur PBCRs were for the years 2018–2019 and 2018, respectively. The number of cancer cases and the population of the respective age group (as per age group 0–4, 5–9, …, 70–74, 75+) were entered into Microsoft Excel for each registry. The region-wise number of cancer cases registered was combined, and the population covered by registries of the respective region was merged. The entered data of registries merged region-wise for calculation. The age-standardised incidence rate (ASIR) was calculated using the statistical methods defined by the IARC [27]. For calculating the HNC burden, we have considered the period year 2012–2019, though the data collected are from different periods. The calculations are based on the data available in the particular period. We have taken the period 2012–2019 which is inclusive of different periods from the data reported by the respective registries. As cancer is a chronic disease, its pattern and burden will not change in a short period.

All defined incidents HNC (age group 0–75+) of each registry were taken as numerators, while the estimated population of geographic area from each PBCR for the respective period was taken as the denominator. The analysis has been done using published data year for both numerator and denominator. As per the published data, the total cases with unknown age group were less than 0.2% in the HNC sites. The HNC cases with unknown ages were equally distributed in all the age groups. For calculating the ASIR, first, the age-specific rates for the age groups were calculated by using the number of cases in that age group as the numerator and the population of that respective age group as the denominator. Later, the ASIR per 100,000 population was calculated using the world standard population along with a 95% confidence interval (CI) [27]. The cumulative risk was calculated using the statistical methods defined by the IARC [27] to know the risk that an individual would have of developing cancer in a certain age span if no other causes of death had occurred.

## Results

### The incidence of HNC in India

**Figure 1.**
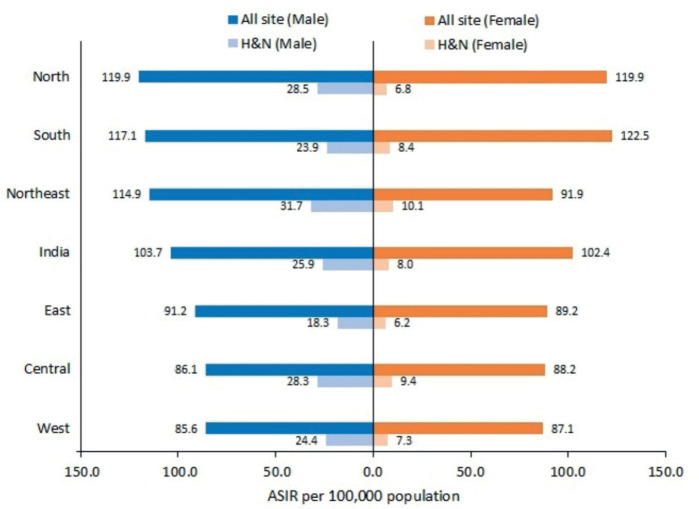
Comparison of all site cancer burden with HNC burden in different regions of India (2012–2019).

The all-site cancer incidence rate was 103.7 and 102.4 per 100,000 population for males and females, respectively. The ASIR for HNC was 25.9 (95% CI 25.7–26.1) and 8.0 (95% CI 7.9–8.1) per 100,000 population for males and females, respectively. The burden of HNC as compared to all-site cancer is mentioned in Figure 1.

### The HNC burden across different age groups and gender

We observed that ASIR for HNC was increasing with age. The HNC burden was the lowest in the age group 0–19 years (0.1–0.3 in males and 0.1–0.2 in females per 100,000 population). For the age group 20–39 years, males residing in the central region had the highest burden of HNC (15.0 per 100,000 population).

For the age group 40–59 years, the northeastern registries reported the highest burden of the disease among males with ASIR 62.6 per 100,000 population; and among females, it was high in the central region with ASIR 20.8 per 100,000 population. The incidence rate of age group 40–59 was in the range of 36.3–62.6 and 12.4–20.8 per 100,000 population in males and females, respectively.

Compared to all other age groups, age 60 and above reported higher ASIR for both males and females. For all registries combined, the overall ASIR for males and females (age 60+ years) was 114.9 and 36.9 per 100,000 population, respectively. In the age group 60+, the highest incidence was reported by registries from the northeastern region for both males and females 155.3 and 46.2 per 100,000 population. The region-wise HNC burden among different age groups is presented in Table 1.

### The HNC incidence in different regions

**Figure 2.**
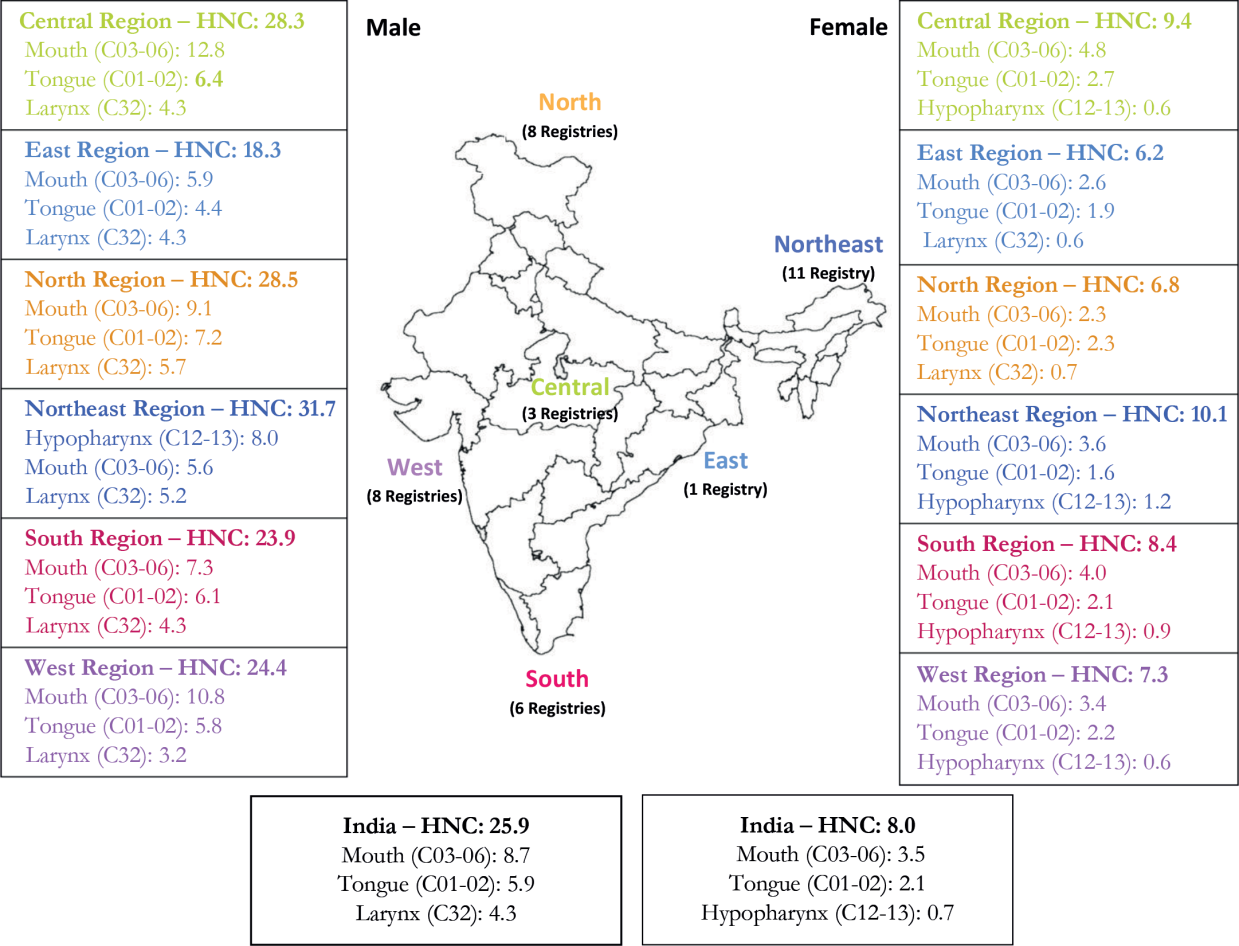
HNC burden along with the first three leading associated sites (2012–2019).

The northeastern registries have reported the highest incidence of HNC in males 31.7 (95% CI 31.1–32.3) per 100,000 population followed by the registries from northern and central India, 28.5 (95% CI 27.9–29.1) and 28.3 (95% CI 27.4–29.2) per 100,000 population respectively. For females, the HNC ASIR among different regions was in the range of 6.2–10.1 per 100,0000 population. The burden of HNC as compared to all-site cancer is mentioned in Figure 2.

### Proportion of HNC to all-site cancer in different regions of India and other countries

As per data analysis of all the registries, among the 218,047 registered cancers in males, 56,046 (25.7%) cases were HNC; while among 217,247 registered cancers in females, 16,795 (7.7%) cases were HNC. Among males, the highest proportion of HNC was reported in the central region (34.1%) followed by western (29.5%), northeastern (27.8%), northern (24.1%), southern (21.0%) and eastern (20.5%) regions. Females had a lower proportion of HNC. Among females, the highest proportion was reported in the central region (10.6%) followed by the northeastern (10.5%), western (8.4%), eastern (6.9%), southern (6.8%) and northern (5.6%) regions.

In other parts of the world, especially developed countries such as USA, the UK and Australia, the proportion of HNC to all-site cancer is much lower than in India; 4.3%, 3.7% and 4.0%, respectively for males while for females the proportion is less than 2%. Moreover, the proportion of HNC to all-site cancer in developing nations including Siraha, Saptari, Dhanusha and Mohattari (SSDM) – Nepal, Columbo-Sri Lanka, Uganda-Kyadondo country (Africa) and Curitiba-Brazil is lower than India for both males and females [28–30]. The proportion of HNC as compared to all-site cancer by regions in India as well as other countries is mentioned in Table 2.

### Leading sites in HNC in different regions of India

Among males, mouth, tongue, larynx, hypopharynx and tonsil were the top five leading cancer sites in the central, eastern, northern, southern and western regions PBCRs while PBCRs in the northeastern region reported hypopharynx as the leading cancer site followed by mouth, larynx tongue and tonsil.

The mouth was the commonest site for HNC for males (ASIR ranges from 5.6 to 12.8 per 100,000 population) and for females (ASIR ranges from 2.3 to 4.8 per 100,000 population). The tongue cancer ASIR per 100,000 population was in the range of 4.4–7.2 and 1.6–2.7 for males and females, respectively.

The site-wise HNC burden with 95% CI for both genders is presented in Table 3. A comparison of the first three associated sites of HNC rate among different regions of India is presented in Figures 2.

### Gender-wise cumulative risk in different regions

In India, one in 33 males and one in 107 females are at risk of developing HNC. The highest cumulative risk was reported among the northeastern region male population (1 in 26 was at risk of developing HNC). This was followed by northern (1 in 30), central (1 in 31), southern (1 in 35), western (1 in 36) and eastern (1 in 47). For other anatomical sites, the cumulative risk was high for mouth cancer followed by tongue.

Region-wise, females from the northeastern region showed the highest cumulative risk (1 in 83), followed by central (1 in 92), southern (1 in 101), western (1 in 118), northern (1 in 128) and eastern (1 in 141). For other anatomical sites, similar to the male population, mouth and tongue cancer cumulative risk was the highest. The cumulative risk for both genders is presented in Table 3.

### Comparison of associated sites of HNC between India and other countries

Overall, India shows higher ASIR for mouth, tongue and hypopharynx cancers compared to the USA, UK, Australia, Uganda-Kyadondo country (Africa) and Brazil as well as SSDM – Nepal and Colombo – Sri Lanka for both genders [28–30].

Mouth cancer ASIR for the Indian male population was 8.7 per 100,000 population which was more or less similar to Columbo – Sri Lanka and SSDM-Nepal, 8.2 and 6.2 per 100,000 population, respectively. The ASIR was much lower for the USA (1.8), Australia (2.1) and UK (2.2) compared to India. For females, the mouth cancer ASIR is 3.5 in India; while it ranges from 0.8 to 1.7 in other countries.

Compared to India where tongue cancer ASIR was reported 5.9 per 100,000 male population, the ASIR for other countries ranged from 1.2 to 4.1 per 100,000 population. For females, the ASIR for tongue cancer ranged from the lowest at 0.6 in Uganda-Kyadondo country (Africa) to the highest in India at 2.1 per 100,000 population. The hypopharynx cancer ASIR for males ranged the lowest from 0.5 in Uganda-Kyadondo country (Africa) to the highest in India 3.1 per 100,000 population; whereas for females, it was the highest in India (0.7) to lowest in SSDM-Nepal and Australia (0.1).

**Figure 3.**
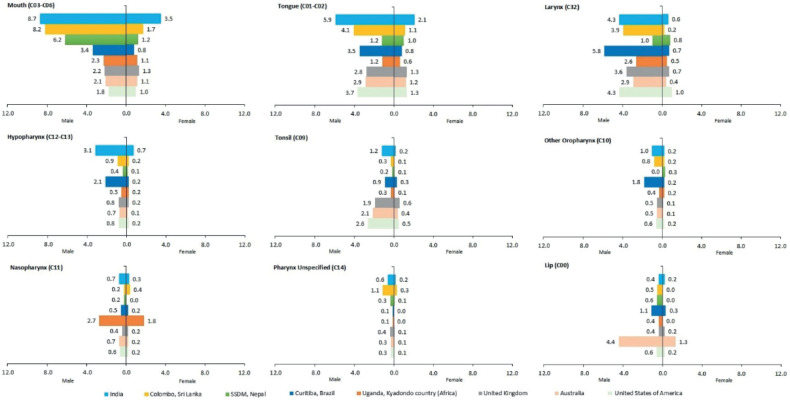
Site-wise comparison of the HNC burden of India with other countries.

There is a variation in ASIR for other HNC sites. The ASIR for laryngeal cancer was reported highest in Brazil (5.8) followed by India and the USA (4.3). The ASIR for cancer of the tonsils was higher in developed nations including the USA (2.6), Australia (2.1) and UK (1.9). Cancer of the nasopharynx was reported highest for both males and females in Uganda, Kyadondo country (Africa) 2.7 and 1.8 per 100,000 population, respectively. Cancer of the lip was reported highest in Australia for both males (4.4) and females (1.3) compared to other countries. The associated site-wise comparison of the HNC burden of India with other countries is illustrated in Figure 3.

## Discussion

The present paper describes the burden of HNC in the different regions of India, based on the published data of 37 PBCRs. As per GLOBOCAN 2020, HNC accounts for 28% of total cancer cases in males and 7% of cases in females in India [1]. Our study found an almost similar proportion of HNC with 26% males and 8% females. In India, the risk of developing HNC is noteworthy in males as well as in females. However, compared to females, males are three times higher in terms of HNC incidence. With regard to age, the incidence of HNC was higher among the age group 40 and above. This finding reflects the need to direct health interventions to address this issue in these age groups. Furthermore, there are limitations in the cancer mortality data in India. It has been reported that insufficient or inaccurate cause-of-death certification and the lack of all-cause mortality data are the reasons behind some registries' low death certificate-only percentages [8]. Not all PBCRs have their mortality data available in the public domain. Hence, we have not analysed the same in this study. However, as per GLOBOCAN 2020, the age-standardised mortality rate of HNC in India for males and females is 14.2 and 4.1 per 100,000 population, respectively [1].

The highest HNC incidence was observed in the northeastern region of India for both males and females. Moreover, when compared to other regions, the incidence of hypopharynx cancer was the highest for both males and females in the northeastern region. The high prevalence of smoking and smokeless forms of tobacco use in the northeast region could be one of the reasons [31].

Compared to other countries such as the USA, UK, Australia, Africa and Brazil overall HNC burden is higher in India. The cancer registries from south-east Asia region such as the Colombo – Sri Lanka as well as SSDM – Nepal have also reported a high burden of HNC [29, 30]. The HNC burden may be higher than the reported data as several registries have reported under-reporting of the cases.

There are several risk factors associated with HNC. The use of tobacco, reverse smoking and alcohol consumption, are the major risk factors for HNC. In addition, poor oral hygiene, micronutrient deficiencies, and human papillomavirus (HPV) infection are reported risk factors for HNC [4, 26, 32]. The variation in ASIR for different HNC sites may be due to risk factor exposure. Mouth and tongue cancer cases are higher in India due to higher tobacco use specifically smokeless form [33]. The regions of India such as central, eastern, northern, southern and western have reported that mouth cancer was the leading cancer site in males followed by tongue, larynx and hypopharynx. Furthermore, the National Family Health Survey-5 also reported that tobacco prevalence is comparatively high in Uttar Pradesh, Bihar, Arunachal Pradesh, Manipur, Meghalaya, Mizoram, Nagaland, Sikkim, Tripura and West Bengal states; it is in the range of 41%–73% in males and 5%–62% in females among these states [34]. Moreover, countries with similar settings such as Sri Lanka and Nepal also reported that higher intake of tobacco and alcohol use increases the risk of HNC [35, 36]. Regarding infectious diseases as a risk factor, a hospital-based study conducted in India reported that HPV prevalence was highest in the oropharynx (41.7%) followed by the hypopharynx (38.9%) and oral cavity (28.4%) [37]. Furthermore, it has been reported that Epstein–Barr virus (EBV) is associated with nasopharyngeal cancer [38]. Conversely, the associated risk factors of HNC in developed countries such as Australia were due to tobacco use especially smoked form as well as alcohol consumption [39]. Additionally, increased sun exposure may be a contributing factor in Australia's higher lip cancer incidence [40].

The burden of associated sites of HNC such as tonsils, other oropharynx, nasopharynx, lip and pharynx unspecified is low in both genders. These cancer sites are considered rare HNC [41]. However, the nasopharyngeal cancer burden in some parts of the northeastern region was high which could be due to high consumption of smoked meat, and EBV infection [42, 43].

In this analysis, we have utilised the data of newly established PBCRs at Varanasi, Uttar Pradesh state and Muzaffarpur, Bihar state [21, 22]. These two states are the most populous states of India. Estimating the burden of the disease was difficult in the past because Bihar and Uttar Pradesh lacked PBCRs. We need to focus on the Uttar Pradesh state of India as the Varanasi registry has reported the highest burden of oral cancer (ASIR: 18.4 per 100,000 population) [21]. The prevalence of tobacco in these states is higher as compared to the national average [34]. The screening by oral visual examination for the high-risk population has resulted in a reduction in oral cancer mortality as per the randomised controlled trial conducted in the Indian population [44]. Furthermore, the cancer registry has played an important role in monitoring the screening programme [45].

Indian PBCRs survival studies have reported low survival in patients with oral cavity cancer [11] as compared to the United States and Europe [46, 47]. There is a dire need to develop and implement cost-effective strategies for cancer prevention and control through early detection and timely treatment. PBCRs will help in the monitoring of these activities. There is a pressing need to strengthen the tobacco control programme in India. The National Tobacco Quit Line services are available in different regions of India, offering cessation services in regional languages [48, 49]. Raising cancer and risk factor awareness in the community will support prevention activities. School education programmes are effective in raising cancer awareness in the community [50].

This study has provided the burden of HNC by utilising the registry data from different regions of India. The burden of HNC varies in different regions of India may be due to the number of PBCRs present in a particular region, coverage of cancer registration and access to diagnostic and treatment facilities. Establishing PBCRs has several challenges such as a lack of cooperation from the cancer-treating centres, inadequate medical records, social stigma about cancer and insufficient funding. To overcome these challenges, PBCR should develop strong institutional relationships and build rapport with data sources as well as interact with patients and their relatives for more accurate information about the disease as well as sociodemographic data [13]. Furthermore, the government authority should make cancer a notifiable disease for a smooth cancer registration process. The results have to be interpreted with caution as there may be underreporting of cases and few registries are recently established. The cancer incidence data for the year 2017–2018 from the PBCRs were not available in the public domain of NCRP at the time of manuscript preparation.

The government of India is playing an important role in strengthening the early detection services of oral, breast, and cervical cancer; however, there are several challenges in implementing these services in the community. Even though the national programme for noncommunicable diseases offers population-based screening, a study suggests low lifetime screening rates (4%–7% in Punjab and Haryana) which is due to logistics and training issues of health workers. Moreover, the uptake of oral cancer screening is extremely poor in both genders [51]. To address this significant public health issue, we must concentrate on finding effective ways to establish an early detection programme. It is reported that the involvement of trained community health workers in screening programme is feasible and effective method [52].

Prominent cancer centres across the nation should step up while assisting capacity building in order to help reduce the unmet needs for cancer control services; for instance, TMCs that are established in various geographical regions of India are playing a key role in developing infrastructure, and capacity building for cancer prevention activities to tackle this important public health problem.

The data generated through this study indicate that the HNC burden is high in India and these cancers should be tackled on a priority basis. As most of these cancers are preventable and the major associated risk factors are tobacco and alcohol consumption, it is recommended that the government should increase awareness of the disease and the harmful effects of tobacco and alcohol consumption among the population. Also, the public health department of the state and central governments should prioritise developing the infrastructure and capacity building in addition to offering early detection services, and facilitating quick access to diagnosis and treatment. Oral cancer screening programme needs to be strengthened. We recommend that medical practitioners as well as oral healthcare providers such as dentists should be trained in diagnosing oral premalignant disorders and oral cancer cases. There should be a hazel-free referral pathway in the healthcare system between healthcare providers and tertiary cancer centres.

## Conclusion

1 in 33 males and 1 in 107 females are at risk of developing HNC in India. Mouth and tongue cancer are commonly prevalent HNC among both genders across India, except males in the northeastern region indicating hypopharynx as a leading cancer site. The burden of HNC is high, especially among the male population. Intensive awareness of risk factors such as tobacco, alcohol and HPV is required. Also, strengthening early detection services is required. Further research and policies on improving the uptake of available cancer screening are required for better cancer control and prevention.

## List of abbreviations

ASIR, Age-standardised incidence rate; CI, Confidence interval; EBV, Epstein–Barr virus; HNC, Head and neck cancer; HPV, Human papillomavirus; IARC, International Agency for Research on Cancer; NCRP, National Cancer Registry Programme; PBCRs, Population-based cancer registries; TMC, Tata Memorial Centre.

## Author contribution

**Sonali Bagal:** Data collection, data analysis, writing – review and editing, quality control.

**Atul Budukh:** Conceptualisation, methodology, writing – original draft, supervision.

**Jarnail Singh Thakur:** Writing – review and editing.

**Tapas Dora:** Writing – review and editing, literature search.

**Burhanuddin Qayyumi:** Data analysis, writing – review and editing, literature search.

**Divya Khanna:** Data analysis, writing – review and editing, quality control.

**Dolorosa Fernandes:** Data analysis writing – review and editing, quality control.

**Priyal Chakravarti:** Literature search, assisted in reviewing and editing, quality control.

**Ravikant Singh:** Writing – review and editing.

**Suvarna Patil:** Writing – review and editing.

**Rajesh Dikshit:** Writing – review and editing.

**Pankaj Chaturvedi:** Writing – review and editing, quality control.

## Conflicts of interest

The author(s) declare that they have no conflict of interest.

## Funding

None.

## Figures and Tables

**Table 1.** HNC burden among different age groups by region and gender (2012–2019).

Age group	Region	Male		Female	
		Number of new cases	ASIR^a^	Number of new cases	ASIR^a^
00–19 (years)	India	184	0.2	90	0.1
	Central	12	0.2	9	0.2
	East	3	0.1	3	0.1
	North	33	0.2	14	0.1
	Northeast	31	0.2	16	0.1
	**South**	**46**	**0.3**	**25**	**0.2**
	West	60	0.2	22	0.1
20–39 (years)	India	6,642	7.7	1,667	2.0
	**Central**	**790**	**15.0**	**158**	**3.1**
	East	213	6.3	63	1.9
	North	1,255	8.1	260	1.8
	Northeast	758	5.1	375	2.6
	South	1,206	6.2	317	1.6
	West	2,421	8.8	493	2.0
40–59 (years)	India	25,813	52.6	7,235	15.8
	**Central**	1,854	58.4	**603**	**20.8**
	East	926	36.3	276	12.4
	North	4,726	57.8	919	12.6
	**Northeast**	**4,962**	**62.6**	1,477	20.2
	South	5,316	45.0	1,787	15.1
	West	8,030	52.1	2,172	15.4
60+ (years)	India	23,407	114.9	7,805	36.9
	Central	1,406	106.5	513	36.9
	East	943	81.1	293	26.9
	North	4,312	127.2	1,108	33.0
	**Northeast**	**4,465**	**155.3**	**1,327**	**46.2**
	South	5,723	114.1	2,386	42.8
	West	6,559	99.1	2,177	31.6

a ASIR: Age-standardised incidence rate per 100,000 population

Bold values represent the region-wise highest ASIR among male and females in a particular age-group

**Table 2.** Proportion of HNC to all-site cancer by regions of India along with other countries by gender.

Region/country (year)	Male			Female		
	All-site cancer	HNC		All-site cancer	HNC	
		Number	%		Number	%
Central (2012–2016)	11,897	4,061	34.1	12,160	1,284	10.6
East (2012–2015)	10,173	2,086	20.5	9,130	634	6.9
North (2012–2019)	42,803	10,325	24.1	41,160	2,301	5.6
Northeast (2012–2016)	36,800	10,216	27.8	30,540	3,195	10.5
South (2012–2018)	58,444	12,290	21.0	66,262	4,516	6.8
West (2012–2018)	57,930	17,068	29.5	57,995	4,865	8.4
India (2012–2019)	218,047	56,046	25.7	217,247	16,795	7.7
SSDM, Nepal (2019)	579	121	20.9	609	40	6.6
Colombo, Sri Lanka (2019)	1,811	275	15.2	2,304	68	3.0
USA (2008–2012)	3,899,202	169,585	4.3	3,656,287	59,788	1.6
UK (2008–2012)	877,231	32,273	3.7	828,286	13,530	1.6
Uganda, Kyadondo country (Africa) (2008–2012)	3,587	186	5.2	4,414	95	2.2
Curitiba, Brazil (2008–2011)	8,624	640	7.4	9,398	154	1.6
Australia (2008–2012)	338,865	13,532	4.0	258,931	4,679	1.8

**Table 3.** Region and gender-wise ASIR of leading HNC sites (2012–2019).

ICD-10	Site	Central	East	North	Northeast	South	West	India
Male								
All	All site (H&N)	28.3	18.3	28.5	31.7	23.9	24.4	25.9
		(27.4–29.2)	(17.5–19.1)	(27.9–29.1)	(31.1–32.3)	(23.5–24.3)	(24.0–24.8)	(25.7–26.1)
		1 in 31	1 in 47	1 in 30	1 in 26	1 in 35	1 in 36	1 in 33
C03-C06	Mouth	12.8	5.9	9.1	5.6	7.3	10.8	8.7
		(12.2–13.4)	(5.5–6.3)	(8.8–9.4)	(5.3–5.9)	(7.1–7.5)	(10.6–11.0)	(8.6–8.8)
		1 in 71	1 in 147	1 in 97	1 in 145	1 in 121	1 in 83	1 in 101
C01-C02	Tongue	6.4	4.4	7.2	5.0	6.1	5.8	5.9
		(6.0–6.8)	(4.0–4.8)	(6.9–7.5)	(4.8–5.2)	(5.9–6.3)	(5.6–6.0)	(5.8–6.0)
		1 in 144	1 in 214	1 in 120	1 in 166	1 in 143	1 in 156	1 in 149
C32	Larynx	4.3	4.3	5.7	5.2	4.3	3.2	4.3
		(3.9–4.7)	(3.9–4.7)	(5.4–6.0)	(4.9–5.5)	(4.1–4.5)	(3.1–3.3)	(4.2–4.4)
		1 in 178	1 in 192	1 in 142	1 in 156	1 in 180	1 in 253	1 in 185
C12-C13	Hypopharynx	2.0	1.7	2.0	8.0	2.6	2.0	3.1
		(1.8–2.2)	(1.5–1.9)	(1.8–2.2)	(7.7–8.3)	(2.5–2.7)	(1.9–2.1)	(3.0–3.2)
		1 in 399	1 in 480	1 in 398	1 in 101	1 in 310	1 in 404	1 in 267
C09	Tonsil	1.1	0.6	1.7	2.4	0.8	0.7	1.2
		(0.9–1.3)	(0.5–0.7)	(1.6–1.8)	(2.2–2.6)	(0.7–0.9)	(0.6–0.8)	(1.15–1.25)
		1 in 727	1 in 1,341	1 in 493	1 in 347	1 in 1,093	1 in 1,205	1 in 721
C10	Other oropharynx	0.5	0.4	1.4	1.4	1.5	0.5	1.0
		(0.4–0.6)	(0.3–0.5)	(1.3–1.5)	(1.3–1.5)	(1.4–1.6)	(0.4–0.6)	(0.96–1.04)
		1 in 1,687	1 in 2,267	1 in 564	1 in 583	1 in 517	1 in 1,564	1 in 775
C11	Nasopharynx	0.3	0.4	0.5	2.3	0.5	0.3	0.7
		(0.2–0.4)	(0.3–0.5)	(0.4–0.6)	(2.1–2.5)	(0.4–0.6)	(0.26–0.34)	(0.66–0.74)
		1 in 2,786	1 in 1,963	1 in 1,940	1 in 375	1 in 1,907	1 in 3,099	1 in 1,298
C14	Pharynx unspecified	0.3	0.5	0.4	1.4	0.4	0.5	0.6
		(0.2–0.4)	(0.4–0.6)	(0.3–0.5)	(1.3–1.5)	(0.3–0.5)	(0.4–0.6)	(0.57–0.63)
		1 in 2,546	1 in 2,010	1 in 2,107	1 in 591	1 in 2,326	1 in 1,645	1 in 1,465
C00	Lip	0.6	0.2	0.6	0.4	0.3	0.5	0.4
		(0.5–0.7)	(0.1–0.3)	(0.5–0.7)	(0.3–0.5)	(0.25–0.35)	(0.4–0.6)	(0.37–0.43)
		1 in 1,240	1 in 4,302	1 in 1,332	1 in 2,296	1 in 2,999	1 in 1,907	1 in 1,991
Female								
All	All site (H&N)	9.4	6.2	6.8	10.1	8.4	7.3	8.0
		(8.9–9.9)	(5.7–6.7)	(6.5–7.1)	(9.7–10.5)	(8.2–8.6)	(7.1–7.5)	(7.9–8.1)
		1 in 92	1 in 141	1 in 128	1 in 83	1 in 101	1 in 118	1 in 107
C03-C06	Mouth	4.8	2.6	2.3	3.6	4.0	3.4	3.5
		(4.4–5.2)	(2.3–2.9)	(2.1–2.5)	(3.4–3.8)	(3.8–4.2)	(3.3–3.5)	(3.4–3.6)
		1 in 174	1 in 340	1 in 384	1 in 234	1 in 204	1 in 254	1 in 244
C01-C02	Tongue	2.7	1.9	2.3	1.6	2.1	2.2	2.1
		(2.4–3.0)	(1.6–2.2)	(2.1–2.5)	(1.5–1.7)	(2.0–2.2)	(2.1–2.3)	(2.0–2.2)
		1 in 334	1 in 430	1 in 381	1 in 501	1 in 390	1 in 398	1 in 403
C12-C13	Hypopharynx	0.6	0.2	0.5	1.2	0.9	0.6	0.7
		(0.5–0.7)	(0.1–0.3)	(0.4–0.6)	(1.1–1.3)	(0.8–1.0)	(0.5–0.7)	(0.66–0.74)
		1 in 1,722	1 in 3,364	1 in 2,025	1 in 623	1 in 1,031	1 in 1,478	1 in 1,207
C32	Larynx	0.6	0.6	0.7	1.0	0.4	0.5	0.6
		(0.5–0.7)	(0.5–0.7)	(0.6–0.8)	(0.9–1.1)	(0.3–0.5)	(0.4–0.6)	(0.57–0.63)
		1 in 1,326	1 in 1,782	1 in 1,157	1 in 820	1 in 2,202	1 in 1,805	1 in 1,455
C11	Nasopharynx	0.1	0.2	0.2	1.1	0.3	0.1	0.3
		(0.05–0.15)	(0.1–0.3)	(0.1–0.3)	(1.0–1.2)	(0.26–0.34)	(0.07–0.13)	(0.28–0.32)
		1 in 9,394	1 in 4,530	1 in 3,632	1 in 852	1 in3,787	1 in 6,429	1 in 2,791
C09	Tonsil	0.2	0.2	0.3	0.6	0.2	0.1	0.2
		(0.1–0.3)	(0.1–0.3)	(0.2–0.4)	(0.5–0.7)	(0.17–0.23)	(0.07–0.13)	(0.18–0.22)
		1 in 5,827	1 in 3,142	1 in 3,092	1 in 1,401	1 in 5,387	1 in 5,861	1 in 3,491
C10	Other oropharynx	0.1	0.1	0.2	0.3	0.2	0.1	0.2
		(0.05–0.15)	(0.04–0.16)	(0.1–0.3)	(0.2–0.4)	(0.16–0.24)	(0.07–0.13)	(0.18–0.22)
		1 in 10,124	1 in 8,048	1 in 3,700	1 in 2,587	1 in 4,333	1 in 6,359	1 in 4,522
C00	Lip	0.3	0.1	0.1	0.3	0.2	0.2	0.2
		(0.2–0.4)	(0.03–0.17)	(0.06–0.14)	(0.2–0.4)	(0.17–0.23)	(0.17–0.23)	(0.18–0.22)
		1 in 3,091	1 in 6,591	1 in 6,203	1 in 2,512	1 in 5,847	1 in 5,268	1 in 4,612
C14	Pharynx unspecified	0.1	0.1	0.1	0.3	0.2	0.1	0.2
		(0.04–0.16)	(0.03–0.17)	(0.06–0.14)	(0.2–0.4)	(0.16–0.24)	(0.07–0.13)	(0.18–0.22)
		1 in 9,951	1 in 6,409	1 in 5,637	1 in 3,430	1 in 4,975	1 in 7,284	1 in 5,516

ASIR: Age-standardised incidence rate per 100,000 population, 95% CI (in parenthesis) and cumulative risk (0–74 age group)
